# Detection of Plasmid-Mediated Colistin Resistant *mcr-1* Gene in *Escherichia coli* Isolated from Infected Chicken Livers in Nepal

**DOI:** 10.3390/ani10112060

**Published:** 2020-11-07

**Authors:** Sayara Bista, Upendra Thapa Shrestha, Binod Dhungel, Pragya Koirala, Tulsi Ram Gompo, Nabaraj Shrestha, Nabaraj Adhikari, Dev Raj Joshi, Megha Raj Banjara, Bipin Adhikari, Komal Raj Rijal, Prakash Ghimire

**Affiliations:** 1 Central Department of Microbiology, Tribhuvan University, Kirtipur, Kathmandu 44618, Nepal; saybista@gmail.com (S.B.); upendrats@gmail.com (U.T.S.); bwith.binod@gmail.com (B.D.); adhikarinaba13@gmail.com (N.A.); joshi_devraj@hotmail.com (D.R.J.); banjaramr@gmail.com (M.R.B.); prakashghimire@gmail.com (P.G.); 2 Central Veterinary Laboratory Ministry of Agriculture, Land Management and Cooperatives, Government of Nepal, Tripureshwor, Kathmandu 44618, Nepal; paggya2000@gmail.com (P.K.); tulsigompo@gmail.com (T.R.G.); naba.shrestha@gmail.com (N.S.); 3 Centre for Tropical Medicine and Global Health, Nuffield Department of Medicine, University of Oxford, Oxford OX1 3SY, UK; biopion@gmail.com

**Keywords:** *Escherichia coli*, colistin resistance, MDR, *mcr-1*

## Abstract

**Simple Summary:**

The poultry industry is one of the top agribusinesses in Nepal. However, despite the government’s restriction on the use of antibiotics as growth promotors in animals, the overuse and misuse of antibiotics can be seen all over the country. Such inappropriate use of antibiotics has led to the rise of antibiotic resistance among treatment options for both human and animal pathogens. Several findings suggest the failure of colistin, a polymyxin E antibiotic (once regarded as the last resort drug), in the treatment of human bacterial infections is due to the emergence and spread of the plasmid-mediated colistin resistance gene (*mcr-1*) among Gram-negative bacterial pathogens. The emergence and rapid transfer of resistant strains in poultry farms are associated with unwanted loss of livestock, economic burden and spread of drug-resistance to other animals, humans and the environment, as well. In this study, we characterized the *mcr-1* gene from infected chicken livers, where prevalence was found to be alarmingly high. This study identifies the result of regulatory failures. Therefore, this report provides valuable reference to the policy makers so that a more effective policy can be formulated and implemented to curb the spread of drug-resistant pathogens.

**Abstract:**

**Background:** Plasmid-mediated resistance to the colistin in poultry is considered as an emerging problem worldwide. While poultry constitutes the major industry in Nepal, there is a paucity of evidence on colistin resistance in *Escherichia coli* isolates causing natural infections in poultry. This study aimed to explore the prevalence of plasmid-mediated colistin resistance gene, *mcr-1* in *E. coli* isolated from liver samples of dead poultry suspected of *E. coli* infections. **Methods:** A total of two hundred and seventy liver samples (227 broilers and 43 layers) from dead poultry suspected of colibacillosis were collected from post-mortem in the Central Veterinary Laboratory (CVL), Kathmandu, between 1 February and 31 July 2019. The specimens were processed to isolate and identify *E. coli*; an antimicrobial susceptibility test (AST) using disk diffusion method was performed with 12 different antibiotics: Amikacin (30 µg), ampicillin (10 µg), ciprofloxacin (5 µg), chloramphenicol (30 µg), cefoxitin (30 µg), ceftazidime (30 µg), ceftriaxone (30 µg), cotrimoxazole (25 µg), gentamicin (10 µg), imipenem (10 µg), levofloxacin (5 µg) and tetracycline (30 µg). Colistin resistance was determined by agar dilution method and colistin-resistant strains were further screened for plasmid-mediated *mcr-1* gene, using conventional polymerase chain reaction (PCR). **Results:** Out of 270 liver samples, 53.3% (144/270) showed growth of *E. coli*. The highest number (54%; 109/202) of *E. coli* isolates was obtained in the liver samples from poultry birds (of both types) aged less than forty days. In AST, 95.1% (137/144) and 82.6% (119/144) of *E. coli* isolates were resistant against tetracycline and ciprofloxacin, respectively, while 13.2% (19/144) and 25.7% (37/144) isolates were resistant to cefoxitin and imipenem, respectively. In the same assay, 76.4% (110/144) *E. coli* isolates were multi-drug resistant (MDR). The phenotypic prevalence of colistin resistance was 28.5% (41/144). In the PCR assay, 43.9% (18/41) of colistin-resistant isolates were screened positive for plasmid-mediated *mcr-1*. **Conclusion:** The high prevalence of *mcr-1* in colistin-resistant *E. coli* isolates in our study is a cause of concern for the probable coming emergence of colistin resistance in human pathogens, due to horizontal transfer of resistant genes from poultry to human isolates.

## 1. Introduction

*Escherichia coli* is a member of *Enterobacteriaceae* family of Gram-negative bacteria and a normal inhabitant of the intestinal tract of humans and animals, although gut commensals can also cause a variety of infections as opportunistic pathogens under favourable conditions [1]. Infections caused by pathogenic *E. coli* (APEC) in poultry are known as colibacillosis and are responsible for sacculitis, pericarditis, peritonitis, salpingitis, synovitis, osteomyelitis, cellulitis, coligranuloma and yolk sac infections in chicken [2]. Most APEC are pathogenic to avian species and pose a low risk to human health. However, some previous studies based on molecular characterization have confirmed the genetic similarity between *E. coli* strains (not specifically APEC) isolated from poultry and those isolated from human infections [3].

All broad-spectrum antibiotics were effective against the infections caused by *E. coli* until the emergence of resistant strains capable of producing resistance enzymes such as beta-lactamases (carbapenemases), i.e., esterases, phophotransferases [4,5]. With the emergence of colistin resistance, a last resort drug to treat life threatening infections (both in humans and animals), concerns about potential transfer of resistance to food chain and to human beings are higher [6]. The burgeoning of antimicrobial resistance (AMR) has compelled the veterinarians to prescribe the drugs which otherwise are not considered as standard regimen of therapy. One such instance is colistin, a polymyxin E antibiotic which is being widely used as a last resort drug for the treatment of carbapenemase, producing multi-drug-resistant Gram-negative bacterial infections, irrespective of neurotoxicity and nephrotoxicity [7,8,9].

The frequently isolated variant of plasmid-encoded mobile colistin-resistant gene (*mcr*), *mcr-1*, was first reported in *E. coli* isolates from livestock and human specimens in China [10]. After an initial report in 2016, *mcr-1* strains have been reported worldwide among several species of *Enterobacteriaceae* [11]. Natural and phenotypic mechanisms were attributed to colistin-resistant strains, the former occurring via mutations of bacterial genomes while the latter was the result of adaptive mechanism [12,13]. The mechanism of horizontal transfer of *mcr-1* was a paradigm shift due to its rapid movement between animals and humans, and vice versa, via mobile genetic elements. Poultry is considered as the major reservoir and good habitat of *mcr-1*-producing organisms that have been isolated from different stages of production and supply chain [1].

While the use of antimicrobials in humans and animals are in comparable proportion, the chance of mutations in animals is higher due to larger animal biomass [14]. Antibiotics in the poultry industry are typically used for growth promotion, prophylactic and therapeutic purposes [15,16], and are administered in inappropriate doses throughout their lifespan [17,18]. Such unsafe drug residues tend to accumulate in various concentrations in edible poultry parts [19]. Continuous exposure to accumulated antibiotics may accelerate the process of antimicrobial resistance by the microbes in the host [20]. The antimicrobial-resistant bacteria originating in poultry populations are transmitted to humans through the environment, food products and direct contact [21], yet the current approaches to tackling AMR suffer from inadequate multi-sectoral and cross-disciplinary efforts embedded in the “one health approach”.

Since poultry remains one of the staple industries in developing countries like Nepal, outbreaks of different sorts of diseases are associated with significant economic burdens [22,23]. In recent years, sudden outbreaks of various avian diseases (mostly collibacillosis) have risen [24]. Treatment regimens against these infections are constantly challenged by unabated AMR. Moreover, most of the diagnoses in Nepal are based on the clinical symptoms and conventional approaches that lack molecular detection and characterization of the causative agent of the disease [22]. These practices have overlooked the precise diagnosis and implementation of the best treatment regimen, which is ultimately worsening the plight of AMR among pathogens [24]. In addition, several other factors such as poultry-rearing conditions, extensive use of drugs and horizontal transfer of resistant genes such as *mcr-1* could be associated with a high burden of colistin resistance among poultries in Nepal. Hence, this study aimed to determine the prevalence of the plasmid-mediated *mcr-1* gene among *E. coli* isolated from infected liver samples of poultry.

## 2. Methods

### 2.1. Sample Collection, Bacterial Isolation and Identification

This study was carried out from 1 February to 31 July 2019. A total of 270 liver samples (227 from broiler and 43 from layers), each obtained from individual dead birds that had died with suspected colibacillosis, were obtained from the postmortem department of the Central Veterinary Laboratory (CVL), Kathmandu. The liver samples processed in this study were from Kathmandu, Lalitpur, Bhaktapur, Dhading, Kavre, Nuwakot and other districts near Kathmandu. More than 70% of samples were from poultry aged less than 40 days. Liver samples were collected from boilers and layers from both small and large flock size (greater than 1500 birds). Demographic information such as geographical district, poultry age, production type and flock size were also recorded during the collection of samples. The samples were collected aseptically and analysed in the Bacteriology Laboratory of CVL as soon as possible after collection. The inoculum obtained was cultured on MacConkey agar (MA) and Eosin Methylene Blue (EMB) agar. After inoculation, the media was incubated for at least 24 h at 37 °C. Colonies showing typical characteristics and morphology of *E. coli* were transferred to nutrient agar and incubated at 37 °C for 24 h. *E. coli* was confirmed by conventional biochemical tests such as indole methyl red, Voges Prausker, citrate utilization test, urease test, oxidative-fermentative test, triple sugar iron test and typical greenish metallic sheen on EMB agar [25,26].

### 2.2. Antimicrobial Susceptibility Testing

All *E. coli* isolates were subjected for antimicrobial susceptibility testing (AST) using the modified Kirby-Bauer disk diffusion method as recommended by the Clinical and Laboratory Standards Institute (CLSI-2017) guidelines using Mueller Hinton Agar (MHA) [27]. The twelve antibiotics used in this study were amikacin (30 µg), ampicillin (10 µg), ciprofloxacin (5 µg), chloramphenicol (30 µg), cefoxitin (30 µg), ceftazidime (30 µg), ceftriaxone (30 µg), cotrimoxazole (25 µg), gentamicin (10 µg), imipenem (10 µg), levofloxacin (5 µg) and tetracycline (30 µg) (Hi-media Laboratories Pvt. Limited, Bombay, India). After inoculation and incubation, the bacterial growth was examined, the zone of inhibition (ZOI) was measured and the results were interpreted as sensitive, resistant and intermediate by comparing with a standard chart as described by CLSI guidelines. All intermediates were also included in the resistant group. Those isolates which were non-susceptible to at least one antibiotic from three or more different classes were reported as multi-drug resistant (MDR) [28].

### 2.3. Determination of Minimum Inhibitory Concentration (MIC) of Colistin

Minimum Inhibitory Concentration (MIC) of colistin was determined by the agar dilution method following CLSI-2017. Different concentrations of colistin ranging from 4 to 32 mg/L were prepared in the agar medium. The inoculums were applied rapidly to the agar surface and the plates were incubated at 37 °C for up to 18 h. The MIC end point was determined as the lowest concentration of antibiotics that completely inhibits the visible growth [29]. According to the CLSI guidelines, isolates with a MIC of ≤4 μg/mL were considered colistin susceptible while MIC of >4 μg/mL were considered colistin resistant.

### 2.4. Quality Control

Each batch of media and reagents was subjected to sterility and performance testing. In AST, the control strains of *E. coli* ATCC 25922 were used as quality control.

### 2.5. Extraction of Plasmid DNA and PCR Amplification of Colistin Resistance Gene (mcr-1)

Plasmid DNA was extracted from *E. coli* isolates by alkaline lysis method [30], and the presence of plasmids was confirmed on 0.8% agarose gel electrophoresis at 120 V for 1 h with ethidium bromide (EtBr) with a concentration of 0.1 µg/mL. A set of primer pairs CLR5-F (5ʹ-CGGTCAGTCCGTTTGTTC-3ʹ) and CLR5-R (5ʹ-CTTGGTCGGTCTGTAGGG-3ʹ) were used for the amplification of *mcr-1*. The amplification was performed in a thermocycler, Prime GeNeiTM (Bangalore, India). The PCR reaction mixture for the *mcr-1* amplification was carried out in a final volume of 25 µL (21 µl of 1× Qiagen Master Mix, 3 µL of template plasmid DNA, 0.5 µL forward and 0.5 µL reverse primer of *mcr-1*, as mentioned above). Amplification was performed following standard methods. The PCR amplification cycle was run, initial denaturation at 95 °C for 15 min, 30 cycles with denaturation at 94 °C for 1 min, annealing at 57 °C for 1.5 min and extension at 72 °C for 1 min, and final extension of 10 min at 72 °C. The amplified products were fractionated by electrophoresis through 1.2% Agarose gel visualized by staining with EtBr. The presence of the *mcr-1* was confirmed by comparing with a positive control and 100 bp DNA Ladder, Thermo Fisher Scientific in the gel run [31,32].

### 2.6. Statistical Analysis

Data were entered and analysed using IBM SPSS Statistics for Windows, Version 24.0, Armonk, NY: IBM Corp, Chicago, IL, USA. Descriptive and inferential statistics were analysed using Chi-square (χ2) test. A *p*-value of <0.05 was considered as statistically significant.

### 2.7. Ethics Approval and Consent to Participate

This study was reviewed by Institutional Review committee, Institute of Science and Technology, Tribhuvan University, Kirtipur, Kathmandu, Nepal. No human sample was involved in this study. Prior to the collection of Liver samples, permission was obtained from the Post-mortem Department, Department of Livestock Services, Central Veterinary Laboratory (CVL), Ministry of Agriculture, Land Management and Cooperatives, Government of Nepal.

## 3. Results

### 3.1. Distribution of E. coli

**Figure 1:**
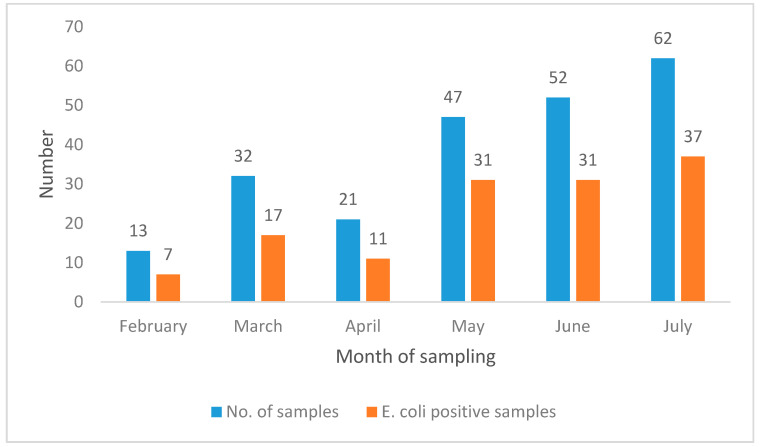
Month-wise distribution of *E. coli* isolated in broiler liver samples.

Out of 270 liver samples, 53.3% (144/270) were positive for *E. coli* and 46.67% (126/270) showed no growth. During the six-month study period from 1 February to 31 July 2019, the highest number of *E. coli* positive samples were isolated from broilers in May (66%; 31/47), June (59.6%; 31/52) and July (59.7%; 37/62) (Figure 1).

**Figure 2:**
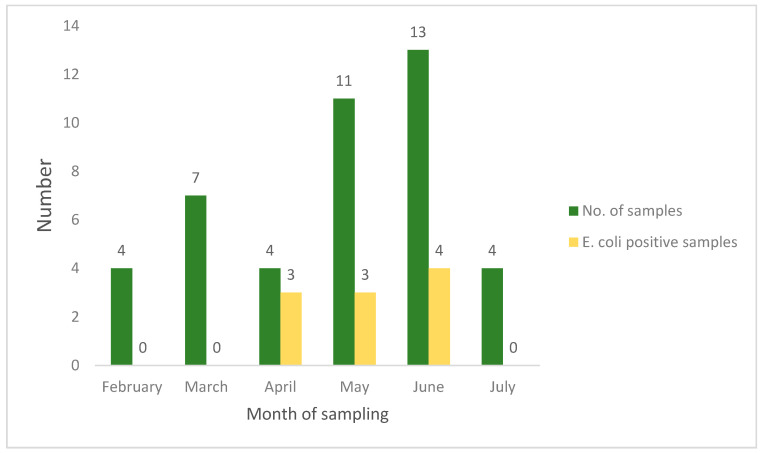
Month-wise distribution of *E. coli* in liver samples from layers.

Similarly, the highest number of *E. coli* was isolated from layers in April (75%; 3/4), followed by the months of May and June (Figure 2).

**Figure 3:**
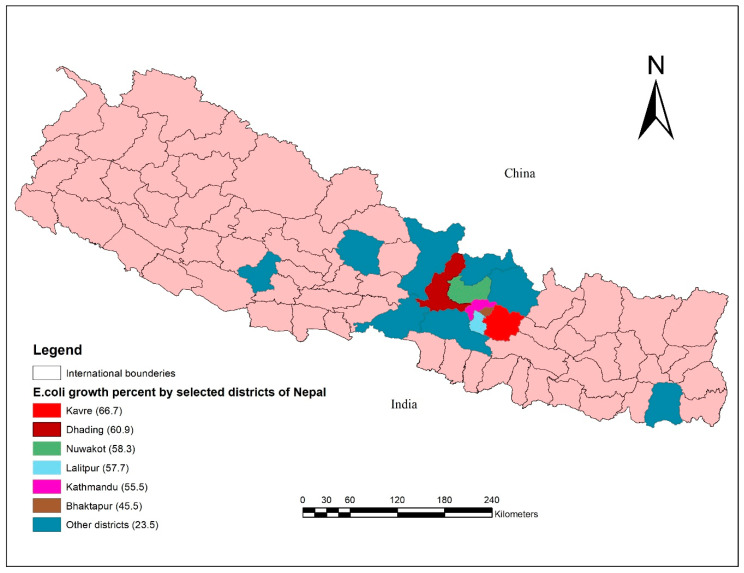
Isolation of *E. coli* in poultry liver samples collected in different districts of Nepal.

The highest number of *E. coli* positive samples was isolated in samples collected from Kavre District (66.7%; 8/12) followed by Dhading District (60.9%; 14/23) and Nuwakot District (58.3%; 7/12) (Figure 3).

From a total of 270 samples, growth of *E. coli* was observed in 144. The highest number (54%; 109/202) was obtained in the liver samples from poultry aged less than 40 days; however, this finding was not statistically significant. There was a significant association concerning the flock size: More than two thirds of the samples from large poultry flocks (>1500) were positive for *E. coli* (74.4%; 67/90), while less than half (42.8%; 77/180) of the samples from small poultry flocks (size < 1500) were positive. Higher prevalence of *E. coli* was observed in broilers (58.60%; 133/227) compared to layers, a finding that was also statistically significant (*p* < 0.05) (Table 1).

### 3.2. Antibiotic Susceptibility Pattern

Among the 12 different antibiotics evaluated, *E. coli* isolates were most frequently resistant to tetracycline 95.1% (137/144) and less resistant to cefoxitin (13.2%; 19/144). A total of 76.4% (110/144) isolates were MDR (Table 2).

### 3.3. Determination of MIC and Colistin-Resistant E. coli Isolates

The MIC value of *E. coli* isolates ranged from 4 to 32 µg/mL. The number of isolates and MIC to colistin is represented on Table 3.

### 3.4. Prevalence of mcr-1 among Colistin-Resistant E. coli Isolates from Poultry Infected Livers

**Figure 4:**
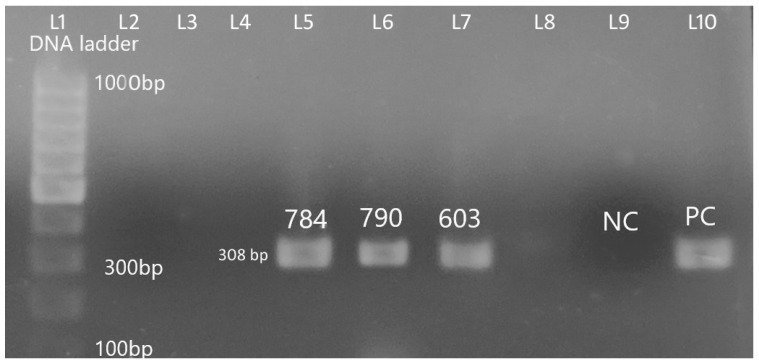
Amplification of plasmid-mediated *mcr-1* gene from different *E. coli* isolates with positive and negative controls (Lane 1, marker (GeneRuler 100 bp DNA Ladder, Thermo Fisher Scientific), Lane 2–Lane 4, no amplifications; Lane 5–Lane 7, positive amplification; Lane 8, no amplification; Lane 9, negative control; Lane 10, positive control).

Forty-one colistin resistant *E. coli* isolates were further screened for plasmid-mediated *mcr-1* using conventional PCR. In the PCR assay, 43.9% (18/41) colistin-resistant *E. coli* isolates were positive for *mcr-1* (Figure 4).

## 4. Discussion

This study aimed to detect plasmid-mediated colistin-resistant *mcr-1* in colistin-resistant *E. coli* strains isolated from poultry suspected to have died with colibacillosis.

Colibacillosis is a major cause of morbidity in poultry [33]. Out of 270 liver samples, 53.3% (144/270) were reported to be infected with *E. coli*, which is similar to a study published in Nigeria reporting 43.3% prevalence of *E. coli* [34]. However, a study from Korea reported prevalence of 4.9% *E. coli* infection in chicken [35]. Growth of *E. coli* in this study was lower than in a previous similar study conducted at Chitwan, a district of Nepal [24]. This diversity may be attributed to differences in geographic location, climate, breeding and growth conditions.

Poultry health management along with biosecurity measures are the emerging issues which can have a major impact on transmission to humans [36]. In our study, the prevalence of *E. coli* isolates was higher (range: 45.5% in Kathmandu to 66.7% in Kavre) compared to the earlier report (33.3%; 60/180) in the four major districts of Nepal: Kathmandu, Bhaktapur, Lalitpur and Kaski [37]. This difference in prevalence rate may have occurred due to the type of poultry and density of flocks included in the studies. Moreover, the greater the flock size, the greater the chance of *E. coli* infections in poultry [38].

There was no significant difference between the growth of *E. coli* and the age of poultry. A slightly higher rate of *E. coli* infection was observed in the poultry ≤40 days old. While there were higher numbers of *E. coli* isolated in younger birds, this finding was not statistically significant and is likely due to sampling bias. Normally, broilers aged 40–42 days (six weeks) are ready for slaughter and marketing in our national context. A higher infection rate before 40 days might have a huge impact on the commercial market of poultry. A higher chance of extensive use of antibiotics in infections occurring after 40 days just before marketing could lead to consumption of meat with high-dose antibiotics. This is a leading cause of the emergence of antibiotic-resistant strains. The highest number of *E. coli* was isolated from flock sizes greater than 1500 (74.4%; 67/90), which is similar to a previous report from Sudan [39]. A higher prevalence of *E. coli* in larger flocks may be due to poor environmental hygiene and sanitation, including frequent faecal contamination of feeds, water, eggs and delay in collection of dead birds [40].

This study reported a higher percentage of *E. coli* in broilers (58.6%; 133/227) than in layers (25.6%; 11/43), which is similar to previous reports from Bangladesh [41] and Sudan [39]. Similar findings were reported from South Korea [42] and the Netherlands [43]. The difference in prevalence among different types of poultry could be attributed to the housing conditions where broilers are reared in semi-intensive systems while the intensive systems are used for layers [39,44]. In addition to this, studies have reported broilers to have comparatively lower immunity than layers [45]. In addition, due to the popularity of broiler poultry farms, there is a higher number of broiler poultry farms in Nepal, which may have led to larger flock sizes, accelerating the spread of pathogens, the use of antimicrobials and consequently the emergence of antimicrobial resistance.

A higher rate of colibacillosis was found during the month of May in this study. The annual technical report of the CVL also suggests historical evidence of a higher rate of colibacillosis in the months of April and May [46]. This could be due to the beginning of the rainy season, which could aggravate the poor hygiene, sanitation and contamination. This study only collected data for February to July and thus does not represent the every season of the year. While the current study was not intended to determine the prevalence or evaluate risk factors for positive samples, the differences may be due to a number of factors including farm size, flock characteristics, farm management, biosecurity practices and climate. Future epidemiological studies should be conducted to explore factors associated with this prevalence, including the potential measures for preventing infectious diseases such as colibacillosis among poultries.

Antibiotic susceptibility profiles of *E. coli* in this study are consistent with previous studies reported from Bangladesh [47], the Netherlands [48] and Nepal [49]. While tetracycline was not in use as a treatment options against *E. coli* infections in poultry farms, high resistance seen towards this drug could be attributable to the misuse of tetracycline as growth promoters [46]. The high sensitivity against cefoxitin could be due to its infrequent use in poultry farms. In this study, 76.4%% of isolates were found to be MDR, which is similar to a report published in the Netherlands [50] and a recent study in Nepal [51]. When compared with previous studies, the finding of this study indicates the persistence (unabated rate) of antibiotic resistance over the period of time [37,51].

This may be due to excessive and inappropriate use of antibiotics for growth promotion and prevention of diseases, which are available over the counter in Nepal [52]. This can further trigger the mechanisms that lead to the emergence of drug-resistant strains of *E. coli*.

Poultry products along with their complex supply chain (farms, transportation to slaughterhouse, the slaughter chain, processing plant, human handling, etc.) facilitate the horizontal exchange of antimicrobial-resistant genes between animals and humans, and vice versa, via mobile genetic elements [53,54]. Moreover, the environment, pets and wildlife are also factors that influence the transmission of AMR between the different reservoirs [54,55].

Similar to our findings, an MIC value of colistin ranging from 4 mg/L to 32 mg/L was reported from Brazil [56] and many other countries [57]. Elevated MIC of colistin in this study is probably due to the continuous exposure of *E. coli* to colistin as a result of its extensive use in feed additives and treatment therapies. The difference in the range of MIC values for colistin may be due to differences in doses of colistin in different countries.

In this study, we screened for *mcr-1* gene among phenotypically colistin-resistant isolates. Eighteen of the colistin-resistant *E. coli* isolates were *mcr-1* positive *showing* MICs for colistin ranging from 4 to 32 mg/L. High 307 prevalence (43.9%; 18/41) of *mcr-1* in our study is similar to other reports from China [58] and Pakistan [59]. Our study does differ slightly from the first report on prevalence of mcr-1 in Nepal, which showed a prevalence rate of 22.8% in colistin-resistant *E. coli* isolates obtained from marketed poultry meat [37]

### Strengths and Limitations

The study serves as a reference tool in epidemiological studies of *mcr-1* genes isolated from poultry in Nepal, although with some limitations. Firstly, we could not confirm colibacillosis in infected samples due to the lack of histological and molecular investigations. In addition, performing nucleotide sequencing to confirm APEC among *E. coli* isolates was beyond the scope of this study. Secondly, the infected livers were analyzed only for bacteriological investigation, so other probable pathogens, such as virus, fungi and parasites were not explored. Thirdly, due to the unavailability of required materials and advanced facilities, including molecular screening of drug-resistant pathogens, we relied on the agar-dilution method in AST, although CLSI has already abrogated this method as a standard tool for the detection of colistin resistance. While this study was a recent one in the context of Nepal, detection of other variants of *mcr* genes (*mcr-1* to *mcr-10*) and whole genome sequencing could be recommended for future studies.

## 5. Conclusions

The high prevalence of colistin-resistant *E. coli* among poultry isolates in this study could be due to the extensive use of colistin in poultry feeds. Moreover, the presence of *mcr-1* in *E. coli* poses a potential threat of spreading colistin-resistant genes to human pathogens, contributing to the emergence of resistance to the last resort of antimicrobials in humans. Therefore, awareness programmes, including the implementation and regulation of strict polices on poultry management and biosecurity measures in addition to surveillance of colistin resistance in poultry, are essential to reduce the inappropriate use of this drug.

## Figures and Tables

**Table 1:** Growth of *E. coli* in the poultry liver samples according to age of dead poultry, flock size and type of poultry.

**Age (Days)**	***E. coli***	**No Growth**	**Total**	***p*-Value**
≤40	109(54%)	93(46%)	202(74.8)	0.72
>40	35(51.5%)	33(48.5%)	68(25.2%)	
Total	144	126	270(100%)	
**Flock Size**	***E. coli***	**No Growth**	**Total**	***p*-Value**
>1500	67(74.4%)	23(25.6%)	90(33.3%)	0.0001
≤1500	77(42.8%)	103(57.2)	180(66.7%	
Total	144	126	270(100%)	
**Type of Poultry**	***E. coli***	**No Growth**	**Total**	***p*-Value**
Broiler	133(58.6%)	94(41.4%)	227(84.1%)	0.0001
Layer	11(25.6%)	32(74.4%)	43(15.9%)	
Total	144	126	270 (100%)	

**Table 2:** Antimicrobial susceptibility pattern of *E. coli* isolates from colibacillosis-related dead poultry (n = 144).

Antibiotic Category	Antibiotics	Susceptibility Pattern			
		Resistant		Sensitive	
		N.	%	N.	%
Aminoglycosides	Amikacin	103	71.5	41	28.5
	Gentamicin	110	76.4	34	23.6
Penicillins	Ampicillin	88	61.1	56	38.9
Cephamycins	Cefoxitin	19	13.2	125	86.8
3rd generation cephalosporins	Ceftriaxone	46	31.9	98	68.1
Phenicols	Chloramphenicol	84	58.3	60	41.7
Fluoroquinolones	Ciprofloxacin	119	82.6	25	17.4
	Levofloxacin	110	76.4	34	23.6
Folate pathway inhibitors	Cotrimoxazole	98	68.1	46	31.9
Carbapenems	Imipenem	37	25.7	107	74.3
Tetracyclines	Tetracycline	137	95.1	7	4.9

**Table 3:** Colistin susceptibility pattern of *E. coli* at different concentrations.

Organism	Number	Concentration of Colistin				Total
		4 mg/L	8 mg/L	16 mg/L	32 mg/L	
*E. coli*	144	14 (34.1%)	10 (24.4%)	8 (19.5%)	9 (22%)	41 (28.5%)
